# Risk Factors Associated with Discordant Ki-67 Levels between Preoperative Biopsy and Postoperative Surgical Specimens in Breast Cancers

**DOI:** 10.1371/journal.pone.0151054

**Published:** 2016-03-08

**Authors:** Hyung Sun Kim, Seho Park, Ja Seung Koo, Sanghwa Kim, Jee Ye Kim, Sanggeun Nam, Hyung Seok Park, Seung Il Kim, Byeong-Woo Park

**Affiliations:** 1 Department of Surgery, Yonsei University College of Medicine, Seoul, Republic of Korea; 2 Frontier Research Institute of Convergence Sports Science, Yonsei University, Seoul, Republic of Korea; 3 Department of Pathology, Yonsei University College of Medicine, Seoul, Republic of Korea; School of Medicine, Fu Jen Catholic University, TAIWAN

## Abstract

**Purpose:**

The Ki-67 labelling index is significant for the management of breast cancer. However, the concordance of Ki-67 expression between preoperative biopsy and postoperative surgical specimens has not been well evaluated. This study aimed to find the correlation in Ki-67 expression between biopsy and surgical specimens and to determine the clinicopathological risk factors associated with discordant values.

**Patients and Methods:**

Ki-67 levels were immunohistochemically measured using paired biopsy and surgical specimens in 310 breast cancer patients between 2008 and 2013. ΔKi-67 was calculated by postoperative Ki-67 minus preoperative levels. The outliers of ΔKi-67 were defined as [lower quartile of ΔKi-67–1.5 × interquartile range (IQR)] or (upper quartile + 1.5 × IQR) and were evaluated according to clinicopathological parameters by logistic regression analysis.

**Results:**

The median preoperative and postoperative Ki-67 levels were 10 (IQR, 15) and 10 (IQR, 25), respectively. Correlation of Ki-67 levels between the two specimens indicated a moderately positive relationship (coefficient = 0.676). Of 310 patients, 44 (14.2%) showed outliers of ΔKi-67 (range, ≤-20 or ≥28). A significant association with poor prognostic factors was found among these patients. Multivariate analysis determined that significant risk factors for outliers of ΔKi-67 were tumor size >1 cm, negative progesterone receptor (PR) expression, grade III cancer, and age ≤35 years. Among 171 patients with luminal human epidermal growth factor receptor 2-negative tumors, breast cancer subtype according to preoperative or postoperative Ki-67 levels discordantly changed in 46 (26.9%) patients and a significant proportion of patients with discordant cases had ≥1 risk factor.

**Conclusion:**

Ki-67 expression showed a substantial concordance between biopsy and surgical specimens. Extremely discordant Ki-67 levels may be associated with aggressive tumor biology. In patients with luminal subtype disease, clinical application of Ki-67 values should be cautious considering types of specimens and clinicopathological risk factors.

## Introduction

Ki-67 is of clinical interest for potential uses in the management of breast cancer patients [1]. It is informative for classification of breast cancer subtypes, may play a predictive role, and is useful in monitoring the response to neoadjuvant therapy [2,3]. At the 13th St. Gallen International Breast Cancer Conference 2013, most of the panel agreed that Ki-67 could be a surrogate marker for the discrimination between luminal A-like and luminal B-like tumors [4]. Although Ki-67 levels of <14% were well correlated with the results of the gene expression analysis, a clear cutoff point for the Ki-67 level for the definition of luminal A or B subtype was not suggested and quality-assured laboratory specific values should be used [4,5].

Clinically, Ki-67 is measured by immunohistochemistry using the MIB-1 antibody. In 2007, the American Society of Clinical Oncology (ASCO) updated its recommendations for the use of tumor markers in breast cancer and pointed out that immunohistochemically detected proliferation markers including Ki-67 should not be recommended for clinical practice because of an insufficient level of evidence and a lack of standardization of assay reagents, procedures, and scoring [6]. Similar to other immunohistochemically detected biomarkers, the measurement of Ki-67 by immunohistochemistry has methodological variability regarding preanalytical, analytical, and postanalytical issues [7]. Among various factors that can affect Ki-67 immunohistochemistry, the type of biopsy may not be an important methodological issue and samples from both core biopsy and surgical resection can be suitable [7].

However, when considering the growth of the clinical importance of Ki-67 and the practical usefulness of neoadjuvant therapy, the type of specimens used to evaluate Ki-67 level can be clinically significant. Several studies reported that the reliability of Ki-67 assessment was inferior in biopsy samples compared to surgical specimens [8–10]. Fewer tumor cells are observed in core biopsy specimens than in surgically-resected specimens [8]. In addition, tissue samples from a core biopsy are usually obtained from near the central area of a tumor mass, even though the peripheral areas of a tumor are more biologically active and highly proliferative [11]. Furthermore, continuous efforts to test intra-institutional validity are critical because the scoring methodology is not yet standardized [12].

The aim of this study was to investigate the distribution and correlation of Ki-67 expression between preoperative biopsy and postoperative specimens. If some cases showed extremely discordant values between the two types of specimens, we aimed to determine which clinicopathological parameters were associated with discordant results for Ki-67 levels.

## Patients and Methods

### Patient selection

A total of 310 patients who underwent definitive surgery for breast carcinoma at the Severance Hospital of Yonsei University College of Medicine, Seoul, Korea between January 2008 and December 2013 were retrospectively selected. All patients in the study cohort had their Ki-67 levels examined using paired preoperative biopsy and postsurgical specimens. Patients who received neoadjuvant chemotherapy or did not undergo evaluation of both preoperative and postoperative Ki-67 levels were excluded. This study was approved by the Institutional Review Board of Severance Hospital, Seoul, Republic of Korea (IRB No. 4-2015-0680). Written informed consent was waived and patient information was anonymized and deidentified prior to analysis.

### Clinicopathological characteristics

Clinicopathological data including expression of estrogen receptor (ER), progesterone receptor (PR), and human epidermal growth factor receptor 2 (HER2) and Ki-67 levels were obtained from the review of medical records and permanent pathology reports.

Tumors with ≥1% nuclear-stained cells by immunohistochemistry using postsurgical specimens were considered positive for ER and PR according to the ASCO/College of American Pathologists (CAP) guidelines [13]. HER2 staining was scored as 0, 1+, 2+, or 3+ according to ASCO/CAP guidelines [14]. In cases with a HER2 2+ result, fluorescence in situ hybridization (FISH) was performed using a PathVysion *HER2* DNA Probe Kit (Vysis, Downers Grove, IL, USA) and *HER2* gene amplification was defined as a *HER2* gene/chromosome 17 copy number ratio ≥2.0 or a case with HER2 gene/chromosome 17 copy number ratio <2.0 but with average HER2 copy number ≥6.0 signals/cell according to ASCO/CAP guidelines [14]. HER2 was considered positive in cases with an immunohistochemistry score of 3+ or gene amplification by FISH.

The Ki-67 levels were immunohistochemically measured in both biopsy and postsurgical specimens using a primary MIB-1 antibody (Dako Denmark A/S, Glostrup, Denmark) by established protocols of the Department of Pathology at our institution. Using a visual grading system by a pathologist (J.S.K) who had specialized experience in breast pathology, the Ki-67 index of preoperative biopsy tissue samples was scored by counting the number of positively stained nuclei and was expressed as a percentage of total tumor cells. Ki-67 levels of postoperative surgical specimens were determined by calculating the percentages of strongly positive-stained cells among 5,000 tumor cells in whole sections, which were mainly located at the tumor periphery.

### Statistical analysis

Differences between groups were evaluated using the chi-square test. Fisher’s exact test was used when appropriate. The independent two-sample *t*-test was used for comparisons of means for continuous numerical data. Comparison of Ki-67 levels between biopsy and surgical specimens was performed with the Wilcoxon matched-pairs signed-rank test. In this study, ΔKi-67 was calculated as postoperative Ki-67 levels minus preoperative Ki-67 levels. The outliers of ΔKi-67 were defined as less than the lower quartile (Q1) of ΔKi-67 minus 1.5 × interquartile range (IQR) or more than the upper quartile (Q3) plus 1.5 × IQR. A logistic regression analysis was used to investigate significant risk factors associated with ΔKi-67 outliers. All statistical tests were two-sided, and *P*-values <0.05 were considered statistically significant. SPSS software version 20.0 (IBM Inc., Armonk, NY, USA) was used for all analyses.

## Results

**Fig 1:**
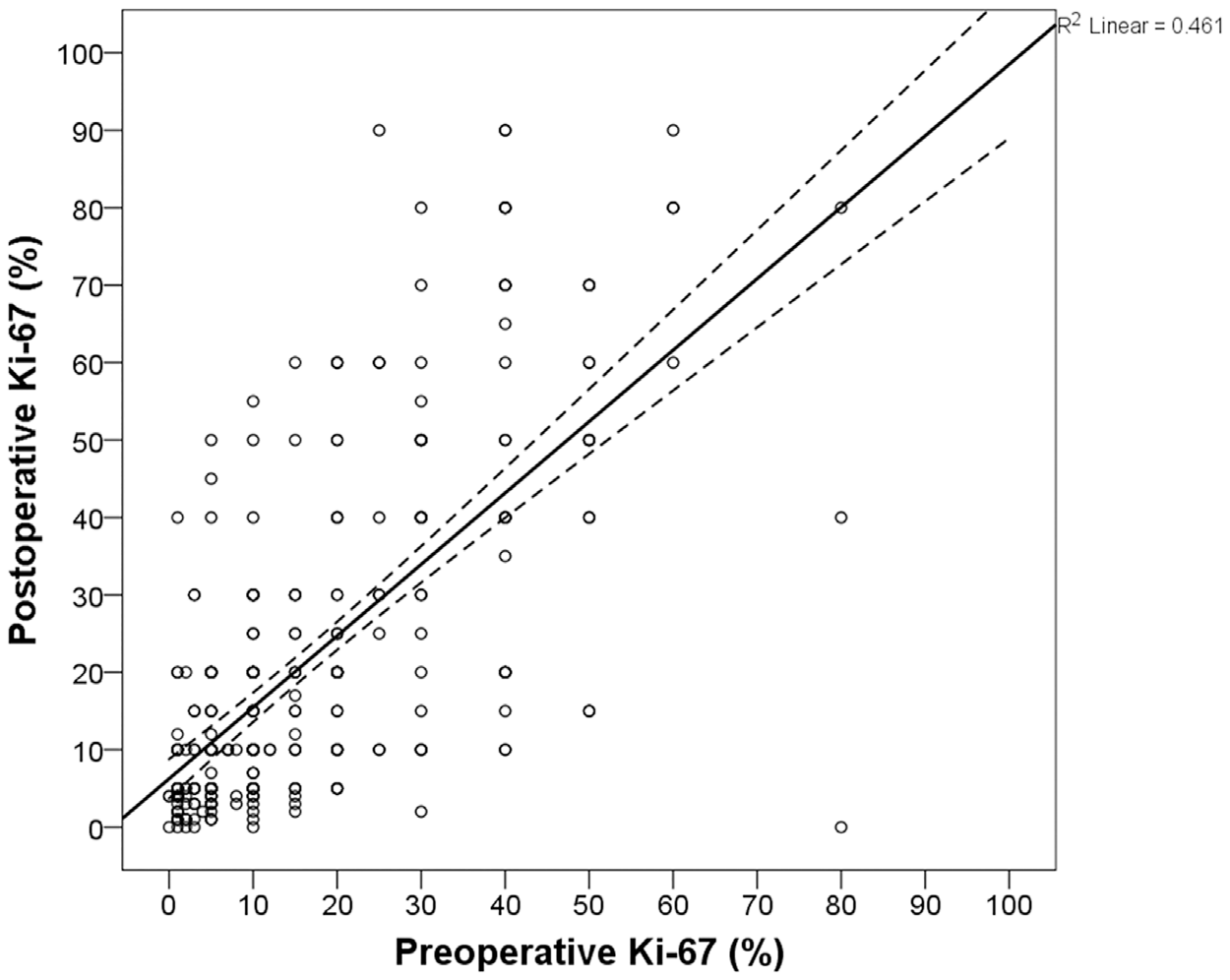
Comparison of Ki-67 expression between preoperative biopsy and postoperative surgical specimens. The solid line presents the best-fit correlation and the dotted lines show the 95% confidence interval.

The median Ki-67 levels from preoperative and postoperative specimens were 10% (IQR, 15; range, 0%–80%) and 10% (IQR, 25; range, 0%–90%), respectively. Wilcoxon matched-pair signed-rank testing revealed a significant association between preoperative and postoperative Ki-67 levels (*P* <0.001). The mean ± standard deviation (SD) of the biopsy and surgical samples were 16.1% ± 15.8% and 21.2% ± 21.5%, respectively. Fig 1 shows the distribution and a positive correlation of Ki-67 levels between the two types of specimens (Spearman’s rho = 0.676; *P* <0.001). When a cutoff value of 14% was applied to define a low or high Ki-67 index, 131 of 177 (74.0%) patients with preoperative low Ki-67 were classified as having postoperative low Ki-67, and 104 of 133 (78.2%) patients with preoperative high Ki-67 were defined as having postoperative high Ki-67 (*P* <0.001).

**Fig 2:**
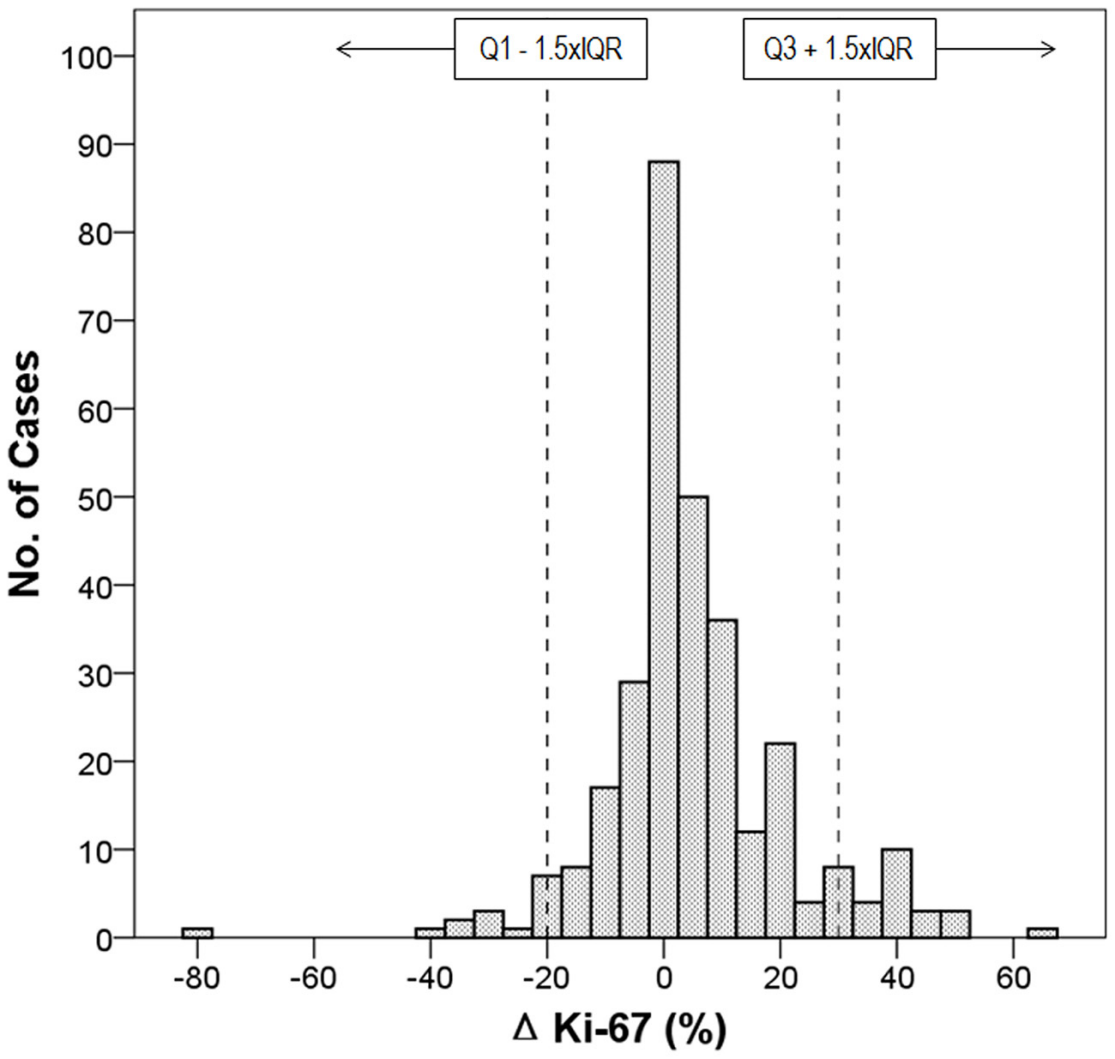
Distribution of ΔKi-67 levels. ΔKi-67 is calculated by postoperative Ki-67 levels minus preoperative Ki-67 levels. Q1, lower quartile; Q3, upper quartile; IQR, interquartile range.

The median value of ΔKi-67 was 2.0 (IQR, 12; range, −80 to 65). The mean difference in proliferation values between biopsy and surgical specimens was 5.0 (SD, 15.8; 95% confidence interval for the mean, 3.25–6.78). In the present study, the outliers of ΔKi-67 were determined as ≤−20 or ≥28. The distribution and outlier range of ΔKi-67 levels are presented in Fig 2. Of 310 patients, 44 were categorized as outliers of ΔKi-67. There was no statistical difference in clinicopathological characteristics except histologic grade between cases with ΔKi-67 ≤−20 (*N* = 15) and ≥28 (*N* = 29). Grade III tumors were frequently categorized as ΔKi-67 ≥28 (*P* = 0.024).

Clinicopathological characteristics of patients classified as ΔKi-67 outliers compared to those within the acceptable range of ΔKi-67 are shown in Table 1. Breast-conservation surgery was performed in 171 patients, and the type of operation was not statistically different between patients classified as ΔKi-67 outliers and those not classified as outliers. Patients younger than 35 years of age and those with tumor size >1 cm, grade III cancers, and ER-negative or PR-negative tumors were significantly more likely to be categorized as ΔKi-67 outliers. Preoperative biopsy tools, histologic type, axillary node status, and HER2 expression were not associated with ΔKi-67 outlier status.

**Table 1:** Clinicopathological characteristics of patients classified as ΔKi-67 outliers.

	Acceptable (%, *N* = 266)	Outlier (%, *N* = 44)	*P*-value
Age (years)			
Mean ± SD	49.8 ± 10.5	49.3 ± 12.2	0.798^a^
≤35	19 (70.4)	8 (29.6)	0.037^b^
>35	247 (87.3)	36 (12.7)	
Preoperative biopsy tool			
Core needle	227 (86.0)	37 (14.0)	0.829
VABB or incisional biopsy	39 (84.8)	7 (15.2)	
Histologic type			
Ductal	237 (85.6)	40 (14.4)	>0.999^b^
Lobular or special	29 (87.9)	4 (12.1)	
Tumor size			
In situ or ≤1 cm	110 (95.7)	5 (4.3)	<0.001
1–2 cm	99 (78.0)	28 (22.0)	
>2 cm	57 (83.8)	11 (16.2)	
Axillary lymph nodes			
Negative	209 (85.3)	36 (14.7)	0.624
Positive	57 (87.7)	8 (12.3)	
Grade			
I/II	209 (91.7)	19 (8.3)	<0.001
III	57 (69.5)	25 (30.5)	
ER			
Negative	58 (72.5)	22 (27.5)	<0.001
Positive	208 (90.4)	22 (9.6)	
PR			
Negative	105 (75.5)	34 (24.5)	<0.001
Positive	161 (94.2)	10 (5.8)	
HER2			
Negative	181 (84.6)	33 (15.4)	0.445
Equivocal	36 (92.3)	3 (7.7)	
Positive	49 (86.0)	8 (14.0)	

SD, standard deviation; VABB, Vacuum-assisted breast biopsy system; ER, estrogen receptor; PR, progesterone receptor; HER2, human epidermal growth factor receptor 2.

^a^Independent samples *t*-test

^b^Fisher’s exact test

Risk factors for extremely discordant values of ΔKi-67 levels were investigated (Table 2). Tumors >1 cm, PR-negative tumors, grade III cancers, ER-negative tumors, and age ≤35 years showed significantly higher odds ratios, in that order. When these parameters were entered into multivariate logistic regression analysis, significant risk factors for outliers of ΔKi-67 were found to be size >1 cm, negative PR, grade III, and age ≤35 years. In our study cohort, 4 of 7 patients with all these risk factors were included among the ΔKi-67 outliers, and among these patients, 3 showed discordantly higher Ki-67 levels in postsurgical specimens than in preoperative biopsy samples.

**Table 2:** Logistic regression models of risk factors associated with classification as ΔKi-67 outlier.

	Univariate			Multivariate^a^		
	OR	95% CI	*P*-value	OR	95% CI	*P*-value
Age (years)						
>35	Ref			Ref		
≤35	2.889	1.178–7.084	0.020	3.290	1.121–9.659	0.030
Preoperative biopsy tool						
Core needle	Ref					
VABB/incisional biopsy	1.101	0.458–2.645	0.829			
Histologic type						
Ductal	Ref					
Lobular or special	0.817	0.273–2.450	0.719			
Tumor size						
In situ or ≤1 cm	Ref			Ref		
>1 cm	5.500	2.101–14.400	0.001	3.773	1.216–11.707	0.022
Axillary lymph nodes						
Negative	Ref					
Positive	0.815	0.359–1.850	0.625			
Grade						
I/II	Ref			Ref		
III	4.825	2.482–9.377	<0.001	2.496	1.093–5.698	0.030
ER						
Positive	Ref			Ref		
Negative	3.586	1.856–6.930	<0.001	0.914	0.354–2.356	0.852
PR						
Positive	Ref			Ref		
Negative	5.213	2.471–11.000	<0.001	3.529	1.394–8.935	0.008
HER2						
Negative/equivocal	Ref					
Positive	0.984	0.431–2.249	0.970			

OR, odds ratio; CI, confidence interval; Ref, reference; VABB, Vacuum-assisted breast biopsy system; ER, estrogen receptor; PR, progesterone receptor; HER2, human epidermal growth factor receptor 2.

^a^Multivariate analysis was conducted using variables that were statistically significant in univariate analysis.

Among 171 patients with hormone receptor-positive and HER2-negative tumors, breast cancer subtypes were compared according to both preoperative and postoperative Ki-67 levels. The cutoff point of Ki-67 <14% was used for the discrimination of the luminal A subtype from the highly proliferative luminal B, HER2-negative subtype. Concordant luminal A and luminal B subtypes were classified in 97 (56.7%) and 28 (16.4%) patients, respectively, regardless of the types of specimens. However, 30 (17.5%) patients whose tumors were categorized as luminal A subtype according to preoperative Ki-67 levels discordantly changed to luminal B subtype according to postoperative Ki-67 levels, and the opposite occurred in 16 (9.4%) patients.

According to the number of our risk factors (size >1 cm, negative PR, grade III, or age ≤35 years), it was explored whether breast cancer subtypes were concordantly or discordantly classified (Table 3). Breast cancer subtype according to preoperative or postoperative Ki-67 levels had discordantly changed in 46 (26.9%) patients. A significant proportion of patients with a discordant subtype had ≥1 of our risk factors (*P* = 0.004) and as the number of risk factors increased, higher proportions of patients showed discordant breast cancer subtypes. Among patients with ≥2 risk factors, 17 (38.6%) cases were categorized into discordant subtypes. Of these, 12 cases changed subtype from preoperative luminal A to postoperative luminal B and 5 changed subtype from preoperative luminal B to postoperative luminal A subtype.

**Table 3:** Association of number of risk factors with breast cancer subtype using preoperative and postoperative Ki-67 levels in 171 patients with hormone receptor-positive and HER2-negative tumors.

Subtype	Number of risk factors			Total	*P*-value
	0 (*N* = 45)	1 (*N* = 82)	≥2 (*N* = 44)		
Concordant	41 (91.1%)	57 (69.5%)	27 (61.4%)	125 (73.1%)	0.004
Discordant	4 (8.9%)	25 (30.5%)	17 (38.6%)	46 (26.9%)	

HER2, human epidermal growth factor receptor 2

## Discussion

In daily clinical practice, one of the major concerns regarding the categorization of breast cancer subtype using immunohistochemical markers is their reliability as a predictive factor, especially in relation to the use of chemotherapy for patients with luminal subtypes [15]. In the First Korean Breast Cancer Treatment Consensus Conference 2014, half of the Korean panelists chose the use of adjuvant chemotherapy for postmenopausal woman with T1c, node-negative, PR-positive, low Ki-67, and grade II disease [16]. Although semiquantitative immunohistochemical expression of PR is also additive to the prognostic information for luminal subtypes [17,18], a current challenge is that the determination of a cutoff point for Ki-67, a single level which has proven prognostic and predictive value, is difficult [15,19].

Clinicians should determine therapeutic modalities by the assessment of biomarkers using preoperative core needle biopsy, postoperative specimens, or infrequently both. However, the concordance rates of Ki-67 levels between core needle biopsy and postsurgical specimens have not been much investigated. Moreover, a few studies have reported that the concordance rates of Ki-67 between the two types of samples were lower than those for ER, PR, or HER2 expression [9,10,20,21]. However, the International Ki-67 in Breast Cancer Working Group commented on the type of biopsy and stated that both core and whole sections were suitable, although some data suggested that scores from whole sections might be higher than those from core biopsies [7]. In the present study, a moderately positive linear relationship was shown (coefficient = 0.676) and Ki-67 levels from surgical specimens were higher (median 2.0; mean 5.0) than those from preoperative biopsy specimens. This suggests that specimens from either biopsy or surgery could be acceptable for the evaluation of Ki-67 but that in a certain group of patients, it might be important to consider the type of specimen with particular caution.

The reason for discordant Ki-67 expression between two specimens may be associated with various methodological issues including sampling problems or tumor heterogeneity [2,7]. Because of its explorative and retrospective nature, although preanalytical variability was not considered in the present study, we hypothesized that extremely discordant Ki-67 levels might be associated with certain clinicopathological parameters and that it might be possible to recommend the evaluation of Ki-67 using both core biopsy and surgical specimens in patients with certain risk factors. As there is no clear definition of extremely discordant values, we used the outliers of ΔKi-67, which constituted 14.2% of the present study population.

Our study revealed that patients with extremely discordant Ki-67 levels between biopsy and surgical specimens were more likely to have poor prognostic factors, which were larger tumor size, negative PR, grade III, or younger age at diagnosis. In a population-based study of Ki-67 using surgical specimens, core biopsy, and tissue microarrays (TMAs), an increase in variability of the difference in Ki-67 between whole sections and TMAs was detected as the Ki-67 average of whole sections and TMAs increased [22]. However, Chen et al. [20] reported that there was no difference in the concordance rate according to tumor stage and that in cases with ER-negative, PR-negative, or grade III tumors, the concordance rate of Ki-67 was rather higher. Different study populations or methodologies may partly explain the different results between studies. Importantly, our explorative study had limitations of interobserver and intraobserver variability for evaluating Ki-67 labelling index since a pathologist (J.S.K) alone had interpreted Ki-67 stained slides. Therefore, our analysis was not confirmative and further independent validation with large samples is necessary.

The clinical implications of Ki-67 levels are of critical importance in hormone receptor-positive and HER2-negative tumors. It has been reported that 14%–21% of tumors classified as luminal A subtype on core biopsy would be discordantly upgraded to luminal B on the basis of surgical specimens [20,22]. Similarly, 17.5% of cases changed from luminal A on biopsy specimens to luminal B on surgical specimens in the present study. Therefore, it has been suggested that repeated Ki-67 assessment on both core needle and open excision biopsies should be performed or that tissue-specific cutoff points of Ki-67 should be applied in clinical practice [20,22]. In addition to prior studies, our results could clarify a subgroup of patients who require particular clinical attention, which were those with ≥1 of risk factors in our study. At present, prognosis according to discordant subtypes was not investigated due to the relatively short follow-up duration of the current study. However, our risk factors were traditional poor prognostic factors; therefore, the benefit and harm of over- or under-treatment according to the discordant classification of breast cancer subtypes should be calculated in the near future.

In conclusion, immunohistochemical Ki-67 expression demonstrated a substantial concordance between preoperative biopsy and surgical specimens. Postoperative Ki-67 levels were likely to be higher than preoperative values. Approximately one-seventh of patients showed extremely discordant Ki-67 levels between the two types of specimens, and risk factors for the outlier values were associated with poor prognostic factors such as larger size, PR-negative expression, grade III disease, and younger age at diagnosis. In patients with luminal HER2-negative tumors, the clinical classification of breast cancer subtype or decision making regarding therapeutic modalities based on the routine pathologically assessed Ki-67 value should be approached with caution considering the types of specimens and the patients’ clinicopathological risk factors.
